# SPR-YOLOv8: A Real-Time Instance Segmentation and Dynamic Size Measurement System for Diamond Particles

**DOI:** 10.3390/s26103004

**Published:** 2026-05-10

**Authors:** Li Wang, Hanwen Niu, Tao Wang, Qiao Wang, Qunfeng Niu

**Affiliations:** 1 School of Electrical Engineering, Henan University of Technology, Zhengzhou 450001, China; hautwangli@haut.edu.cn (L.W.); a13803784692@163.com (H.N.); wqiao2010@zju.edu.cn (Q.W.); 2 Sanmenxia Zhongyuan Measuring Instrument Co., Ltd., Sanmenxia 472000, China; wangtao@tsinghua.org.cn

**Keywords:** instance segmentation, diamond particles, real-time detection, lightweight network

## Abstract

**Highlights:**

**What are the main findings?**

- A lightweight SPR-YOLOv8 instance segmentation model is proposed, which achieves a high mAP@0.9 of 0.861 with only 0.97 M parameters and an inference speed of 500 FPS, effectively balancing accuracy and efficiency for tiny, dense diamond particles.
- The developed Diameter Particle Size Calculation Algorithm (DPSCA) based on segmentation contours reduces the mean absolute percentage error (MAPE) of particle size measurement by over 70% compared to traditional OpenCV methods, demonstrating superior accuracy and robustness.

**What are the implications of the main findings?**

- The proposed integration scheme (SPR-YOLOv8 + DPSCA) provides a practical, high-precision, and real-time solution for automated online inspection in superhard material manufacturing, addressing the industry’s pressing need for efficient quality control.
- The model’s architectural improvements, including the Large Separable Kernel Attention (LSKA) and RepBlock modules, offer a referential lightweight design paradigm for other industrial vision tasks involving small object detection and segmentation.

**Abstract:**

To meet the demand for real-time and accurate diamond particle size measurement in industrial scenarios—where traditional image processing methods lack robustness in complex environments and existing deep learning models struggle to balance accuracy and efficiency—this paper proposes an integrated framework for dynamic segmentation and morphological analysis of diamond particles based on video streams. A fully automated data acquisition system consisting of a high-precision motion stage, an industrial camera, and an optical microscope is first constructed to capture dynamic particle images. Based on YOLOv8n-seg, a lightweight SPR-YOLOv8 instance segmentation model is then developed with three key improvements: a Large Separable Kernel Attention (LSKA) mechanism is introduced into the SPPF module to enhance feature discriminability; the RepBlock module is adopted in the neck network to improve feature fusion efficiency through structural re-parameterization; and a P2 small-object detection head is introduced while large-object detection branches are removed, enabling the model to focus on tiny, densely distributed particles. Finally, a contour-based geometric analysis method is proposed for particle size estimation based on segmentation results. Experimental results show that the proposed model achieves an mAP@0.9 of 0.861 while maintaining a low parameter count (0.97 M) and a high inference speed of 500 FPS. Compared with the conventional OpenCV-based method (CADPS), the proposed DPSCA framework reduces the mean absolute percentage error in particle size measurement by over 70%, while also demonstrating strong accuracy and stability in consecutive-frame tracking. Overall, this study provides a practical and efficient automated inspection solution for online quality control in superhard material manufacturing, and supplementary cross-scale experiments further demonstrate promising robustness on diamond particles beyond the primary 180–250 μm range.

## 1. Introduction

In recent years, the rapid expansion of the new energy and third-generation semiconductor industries has continuously increased the demand for diamond materials. As a representative superhard material, diamond has substantial application value in oil exploration, mining, aerospace, and semiconductor processing. Diamond raw materials are extensively used in a wide range of sectors, including high-precision manufacturing, electronic material processing, and geological exploration, where their particle size and morphology directly influence performance in specific application scenarios.

Previous studies have demonstrated the critical role of diamond particle characteristics in determining the properties of composite materials. Zhao Junfeng reported that, with increasing particle size, the density and thermal conductivity of composites initially increased and then decreased, whereas the coefficient of thermal expansion gradually increased [1]. Similarly, Zhou Hongyu fabricated diamond/Al composites using a liquid–solid separation method and found that diamond particle size significantly affected key thermophysical properties, including density and thermal conductivity [2].

These findings indicate that the size and morphology of diamond particles directly govern the thermal and mechanical performance of composite materials. Variations in particle size can markedly alter material density, thermal conductivity, and thermal expansion behavior. Therefore, accurate measurement of diamond particle size is of considerable engineering importance.

Traditional methods for particle size analysis primarily rely on manual microscopic measurement or laser diffraction analysis. While these methods offer high precision, they suffer from low efficiency and a heavy reliance on manual operation, making it difficult for them to meet the demands of real-time industrial inspection. Early image processing techniques typically employed algorithms such as threshold segmentation or the Hough transform. However, these methods are highly sensitive to variations in lighting conditions and particle agglomeration, resulting in insufficient robustness within complex imaging environments. Liu Xiaomin proposed a contour edge localization method based on the Hough transform to measure parameters such as the size, roundness, and ellipticity of diamond particles [3]. Guo Shuqing utilized a modified Hough transform to achieve the efficient extraction and measurement of diamond particle edge features [4]. While these studies yielded promising results when applied to samples with regular morphologies, their accuracy and generalization capabilities diminished significantly when confronted with images featuring overlapping particles, irregular shapes, or complex backgrounds. Subsequently, machine vision technology was introduced into the field of particle inspection. This technology has significantly enhanced the level of automation; machine vision systems—equipped with optical imaging and image acquisition modules—can now replace manual observation to automatically perform particle recognition and measurement tasks. Its advantages lie in its strong real-time performance, excellent stability, and high repeatability, leading to its widespread application across fields such as industry [5], transportation [6], agriculture [7], and medicine [8]. The application of machine vision to diamond particle detection can significantly boost inspection efficiency and minimize human error. However, such methods typically rely on manually engineered feature extraction algorithms—such as edge, grayscale, or texture features—which struggle to accommodate variations across different sample batches or lighting conditions, thereby lacking both adaptability and deep feature learning capabilities.

In recent years, Convolutional Neural Networks (CNNs) have opened up new technical avenues for particle detection. Deep learning models are capable of automatically extracting multi-scale texture features, enabling high-precision object detection and segmentation even against complex backgrounds. Lin Zhenkun utilized deep learning models to analyze diamond images, achieving efficient and accurate quality assessment and classification [9]. Huang Yeqi et al. proposed a method for inspecting the surface quality of diamond wires based on an improved YOLOv5 model; this approach enabled the efficient detection of surface defects on diamond wires, thereby elevating the standards of production quality control [10]. Furthermore, the YOLO series of models has demonstrated outstanding performance in various scenarios, including agricultural and industrial inspection. For instance, addressing the issues of low accuracy and poor performance in detecting and identifying tomato leaf diseases within natural environments, Hu Yanru proposed a detection model based on an optimized YOLOv8 architecture; this model significantly enhanced recognition capabilities while achieving a more lightweight design [11]. Liu Zifu employed YOLO-based instance segmentation to detect weeds in orchards, thereby boosting the efficiency of farmland management [12]. a lightweight maturity instance segmentation method of cherry tomato with 5 levels, including green, turning, pink, light red and red was proposed based on improved YOLOv8n-Seg model, named as MobileViTv3-SK-WIoU-YOLOv8n-Seg (MSW-YOLOv8n-Seg) [13], while Ranjan Sapkota, through a comparative analysis of YOLOv8 and Mask R-CNN, demonstrated that YOLOv8 offers superior detection accuracy and speed advantages in complex environments [14]. Addressing the challenge of rapidly identifying and locating car keys—which can be difficult for the human eye to discern amidst the visual clutter of a typical home environment—Chen Congming designed a real-time detection system based on the E-YOLOv8n model; this system achieved substantial model lightweighting while simultaneously enhancing performance [15]. To address the limitations of existing deep learning-based algorithms in detecting surface defects on brake pipe ends, a novel lightweight detection algorithm, FP-YOLOv8 [16]. Although two-stage instance segmentation models offer high accuracy, they are characterized by complex architectures and relatively slow inference speeds. Conversely, single-stage YOLO series models excel in real-time performance; however, they still face limitations regarding segmentation accuracy in scenarios involving small-sized, high-density particles. During the multi-scale feature fusion process, these models may suffer from a loss of feature information, resulting in a diminished capacity to accurately delineate object boundaries.

The present study addresses the conflicting requirements of high segmentation accuracy, lightweight deployment, and real-time particle size measurement in industrial diamond inspection. To overcome these challenges, a novel integrated framework is proposed. The main contributions of this study are summarized as follows:

- A lightweight SPR-YOLOv8 segmentation architecture is developed for dense microparticle detection. By incorporating the SPPF_LSKA attention mechanism, the proposed model enlarges the global receptive field and enhances boundary-aware feature representation, thereby improving segmentation robustness under complex illumination conditions and background interference.
- An efficient neck optimization strategy based on RepBlock is introduced. Through structural re-parameterization, the advantages of multi-branch training are retained while inference complexity is significantly reduced, enabling high-speed real-time deployment.
- A P2-2 small-object detection head is proposed for tiny particle perception. By preserving high-resolution shallow features and removing redundant large-object detection branches, the network becomes more suitable for densely distributed diamond particles.
- A DPSCA contour-based particle size measurement algorithm is proposed. This method converts segmentation masks into reliable geometric measurements, enabling dynamic particle size statistics and continuous online monitoring.
- Preliminary cross-scale validation is conducted on additional particle batches (25–40 μm and 250–350 μm). Experimental results indicate that the proposed framework exhibits promising transferability under particle-scale variation, supporting its potential for practical deployment.
- A complete industrial inspection system integrating image acquisition, segmentation, tracking, and measurement is established. The system demonstrates the practical feasibility of intelligent online quality control for superhard material manufacturing.

## 2. Materials and Methods

### 2.1. Data Preparation and System Workflow

The primary diamond particle samples used in this study were provided by Huazhong Jingwei Company in Jiaozuo, Henan, China, with a nominal particle size range of 180–250 μm. In addition, supplementary samples with nominal size ranges of 25–40 μm and 250–350 μm were collected from separate production batches for preliminary cross-scale robustness evaluation.

**Figure 1:**
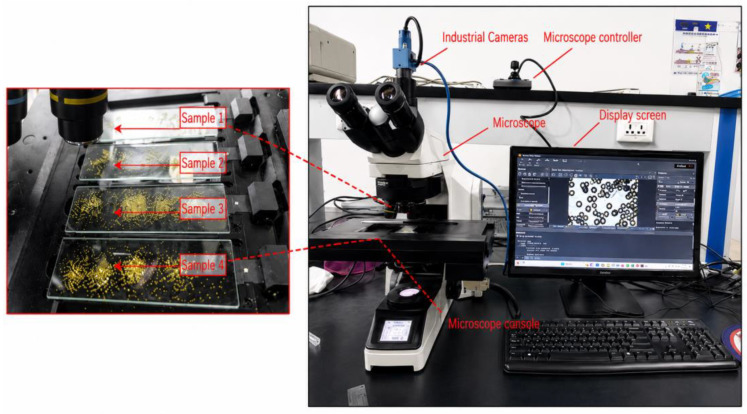
Dynamic image acquisition system.

The dataset was constructed based on a dynamic image acquisition strategy, in which diamond particles were captured as continuous video streams to support real-time analysis. Compared with conventional static imaging, this approach preserves temporal information by recording consecutive frames under controlled motion conditions. A total of 1014 frames with a resolution of 1920 × 1200 were acquired using an integrated imaging system consisting of an optical microscope, a high-frame-rate industrial camera, and a control platform (Figure 1).

Each frame was associated with timestamp information, enabling accurate correspondence between particle positions and their temporal evolution. This dynamic acquisition method effectively captures particle behaviors such as adhesion, rolling, collision, and displacement, thereby reducing measurement errors caused by overlap, occlusion, and boundary ambiguity in single-frame analysis.

**Figure 2:**
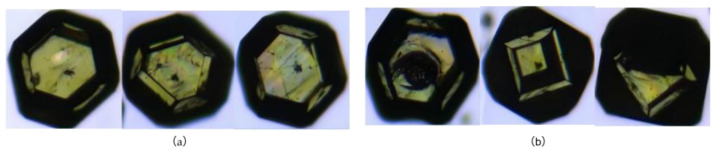
Diamond particle morphology. (**a**) High-mass particles; (**b**) Low-mass particles.

For dataset annotation, an interactive semi-automatic labeling approach (ISAT) is adopted to ensure high-quality instance-level annotations [17]. Each frame is labeled with precise particle masks, and consistent particle IDs are maintained across adjacent frames to preserve temporal continuity. This enables the model to learn both spatial features and motion patterns of particles. The 1014 frames were collected from more than 20 independent video segments acquired across different time points and particle batches, ensuring substantial scene diversity across the dataset. The dataset is divided into training and validation sets with a ratio of 4:1, comprising 811 and 203 frames, respectively, using random frame-wise assignment. To verify the absence of temporal leakage, inter-frame structural similarity (SSIM) was analyzed across three frame pair types: adjacent frames within the training set (SSIM = 0.923 ± 0.041, serving as a within-segment similarity proxy), random cross-set pairs between training and validation frames (SSIM = 0.412 ± 0.089), and random within-training pairs (SSIM = 0.398 ± 0.092). The near-identical similarity between cross-set and random within-training pairs confirms that the random split did not introduce systematic temporal leakage between the two sets. In total, 10,120 high-quality particles (Class A) and 23,277 low-quality particles (Class B) are annotated (Figure 2). During dataset annotation, particles with severely ambiguous or unresolvable boundaries were excluded, since reliable contour delineation could not be consistently achieved through manual labeling. This filtering criterion was based on annotation quality rather than image blur alone. Particles with mild motion blur, moderate defocus, partial overlap, or common illumination variation were retained when their contours remained visually distinguishable. The resulting dataset integrates spatial and temporal information, providing a reliable foundation for YOLOv8-based real-time particle detection, tracking, and dynamic size measurement in video stream scenarios.

**Figure 3:**
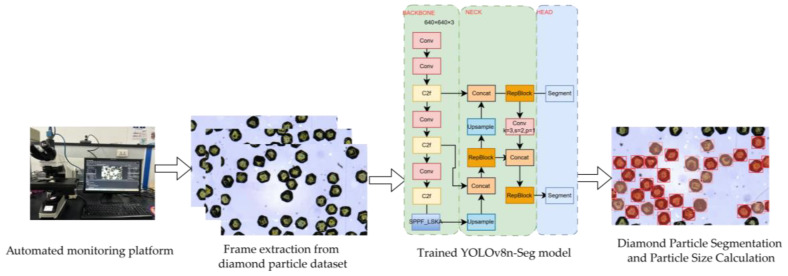
Training methods used. The red box represents the recognized diamond particles.

Based on the aforementioned analysis, a unified technical framework was established, as illustrated in Figure 3. During the training phase, a dedicated diamond particle dataset was constructed using the automated acquisition platform and subsequently used to train the segmentation network.

During the testing and inference phase, the same platform was employed to capture real-time video streams of diamond particles. The trained segmentation network was then deployed together with the proposed particle size measurement and crystal morphology analysis algorithms. Through this integrated system, automatic particle size classification and crystal morphology identification of the test samples were successfully achieved.

Experimental results demonstrate that the proposed model and algorithms can accurately segment diamond particles while reliably identifying their key morphological characteristics. These findings further verify the feasibility of the proposed framework for practical deployment in fully automated particle size inspection and morphology analysis of diamond particles.

### 2.2. SPR-YOLOV8

**Figure 4:**
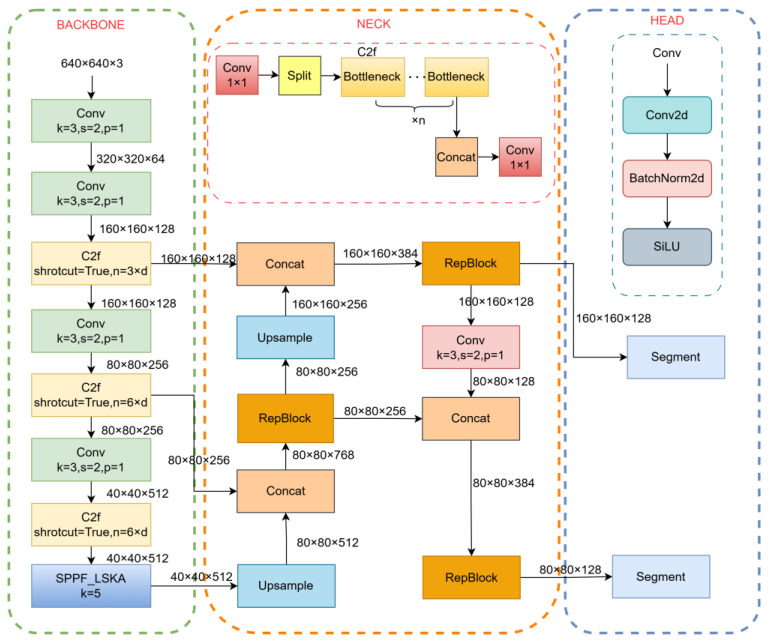
SPR-YOLOv8 model.

YOLOv8 employs a deep residual network for feature extraction and adopts a PAN-based architecture for multi-scale prediction [18]. During feature extraction, multiple downsampling operations are performed to generate high-level semantic feature maps. To better accommodate the characteristics of diamond particles, this study proposes a lightweight segmentation model based on YOLOv8, as illustrated in Figure 4.

Specifically, three major improvements are introduced. First, the SPPF module in the backbone is enhanced by incorporating the Large Separable Kernel Attention (LSKA) mechanism, which strengthens feature extraction capability and improves the representation of particle boundaries. Second, the C2f module in the neck is replaced with a RepBlock module, which utilizes structural re-parameterization to improve feature fusion efficiency while reducing inference overhead. Third, an additional detection head is introduced to enhance small-object segmentation, resulting in a four-head architecture denoted as YOLOv8-P2.

Furthermore, the detection heads designed for large-scale objects (16 × 16 and 32 × 32 feature scales) are removed to reduce computational cost, leading to the final YOLOv8-P2-2 model. This design achieves a better balance between lightweight architecture and detection accuracy for small, densely distributed diamond particles.

#### 2.2.1. SPPF_LSKA: Attention-Enhanced Multi-Scale Feature Aggregation

**Figure 5:**
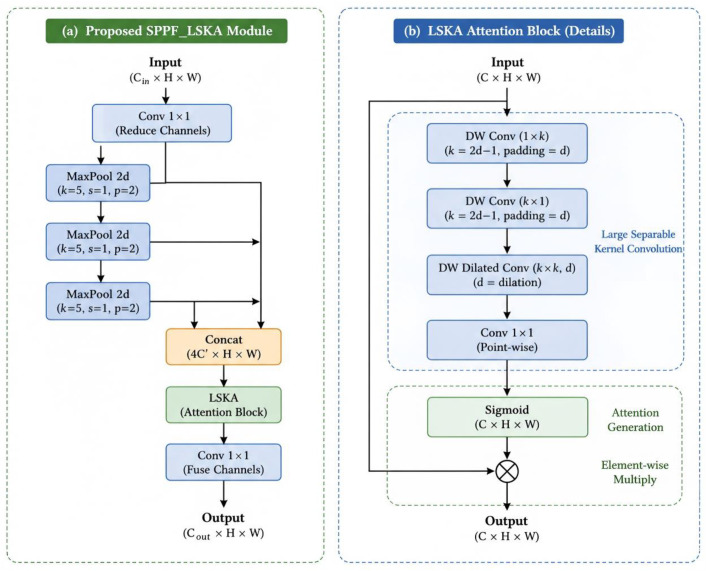
Improved SPPF-LSKA and LSKA structures. (**a**) Detailed architecture of the proposed SPPF _LSKA module; (**b**) the internal LSKA block.

To address the challenges of overexposure and high visual similarity between diamond particles and the background, which often lead to insufficient feature discrimination, an attention-enhanced SPPF module is proposed by integrating the Large Separable Kernel Attention (LSKA) mechanism, termed SPPF_LSKA [19] (Figure 5).

The LSKA module approximates large-kernel convolutions through a series of decomposed operations. Given an input feature map $X\in {\mathbb{R}}^{C\times H\times W}$, the standard large-kernel convolution can be expressed as:

(1) $$Y=X\times K_{k\times k}$$

To reduce computational complexity, it is factorized into separable convolutions:

(2) $$Y=X\times K_{1\times k}\times K_{k\times 1}$$

and further extended with dilation to enlarge the receptive field:

(3) $$Y=X\times K_{1\times k}^{\left(d\right)}\times K_{k\times 1}^{\left(d\right)}$$

Based on the extracted features, an attention map is generated as:

(4) A=σ(Conv(Y))

where σ(⋅) denotes the sigmoid activation function. The final refined feature representation is obtained by:

(5) $$F_{\mathrm{LSKA}}=X⊙A$$

By embedding LSKA into the SPPF structure, the multi-scale pooled features are first aggregated:

(6) $$F_{\mathrm{SPPF}}=\mathrm{Concat}\left(X,\mathrm{MP}(X),{\mathrm{MP}}^{2}(X),{\mathrm{MP}}^{3}(X)\right)$$

and then enhanced via attention:

(7) $$F_{\mathrm{out}}=\mathrm{LSKA}(F_{\mathrm{SPPF}})$$

This design enables the network to capture long-range spatial dependencies and enhances multi-scale contextual aggregation while maintaining low computational cost. As a result, the proposed SPPF_LSKA module significantly improves feature separability under complex imaging conditions. Although it introduces only a marginal increase in the number of parameters, the enhanced representation capability leads to more robust and accurate segmentation performance.

#### 2.2.2. RepBlock: Structural Re-Parameterization for Efficient Feature Fusion

**Figure 6:**
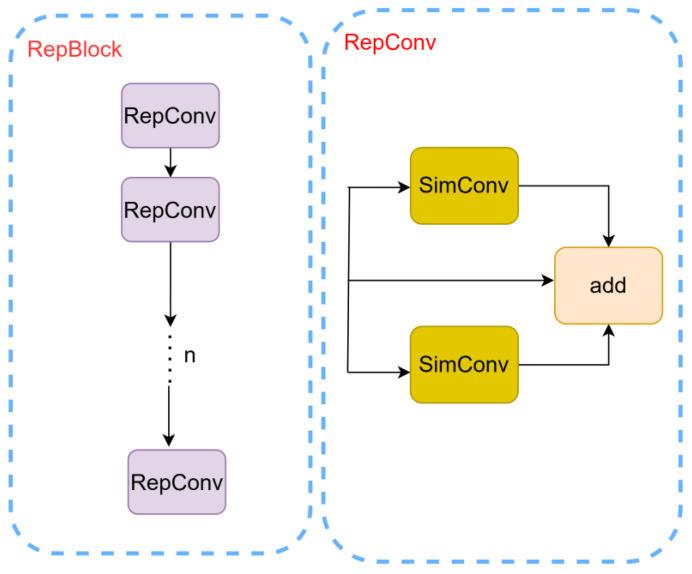
RepBlock module.

To improve the efficiency of multi-scale feature fusion in the neck, the conventional C2f module is replaced with a RepBlock [20], which leverages structural re-parameterization to achieve both enhanced representational capacity and reduced inference cost (Figure 6).

During the training phase, RepBlock adopts a multi-branch architecture composed of multiple convolutional paths and identity mappings. Given an input feature map XXX, the output can be expressed as:

(8) $$Y=\sum_{i=1}^{n}\left(W_{i}\times X+b_{i}\right)$$

where *W_i_* and *b_i_* denote the convolutional kernels and biases of different branches, respectively. A typical formulation includes a 3 × 3 convolution branch, a 1 × 1 convolution branch, and an identity mapping:

(9) $$Y=W_{3\times 3}\times X+W_{1\times 1}\times X+X$$

During inference, these parallel branches are analytically fused into a single equivalent convolution through structural re-parameterization:

(10) $$Y=W_{eq}\times X+b_{eq}$$

where

(11) $$W_{\mathrm{eq}}=\sum_{i=1}^{n}W_{i},\;b_{\mathrm{eq}}=\sum_{i=1}^{n}b_{i}$$

Such a decoupled design allows the network to exploit the representational advantages of multi-branch structures during training while maintaining a lightweight and efficient architecture during inference. Consequently, the proposed RepBlock significantly improves computational efficiency without sacrificing feature representation capability, making it particularly suitable for real-time applications and resource-constrained environments.

#### 2.2.3. P2-Based Lightweight Head for Small Object-Aware Segmentation

Considering that diamond particles are typically small and densely distributed, the original three-scale detection heads in YOLOv8 are insufficient for capturing fine-grained details. To address this limitation, an additional detection head is introduced at the P2 level, forming the YOLOv8-P2 architecture [21]. The P2 feature map, with a higher spatial resolution of 160 × 160, provides richer low-level information, such as edges and textures, which is critical for accurate small-object segmentation.

Furthermore, to achieve a more efficient architecture, the detection heads corresponding to large-scale objects (16 × 16 and 32 × 32 feature scales) are removed, resulting in the YOLOv8-P2-2 model [22]. This modification reduces computational redundancy and memory consumption while preserving the model’s sensitivity to small targets. The improved detection heads are illustrated in Figure 4.

By prioritizing high-resolution feature representation and eliminating unnecessary large-object detection branches, the proposed YOLOv8-P2-2 architecture achieves a more favorable balance between accuracy and efficiency. It demonstrates superior performance in scenarios involving dense and fine-grained particle distributions while maintaining real-time inference capability.

## 3. Results

### 3.1. Model Training Platform and Evaluation Metrics

All models were trained on a 64-bit operating system using a hardware platform equipped with an Intel i9-13900KF CPU, 64 GB RAM, and an NVIDIA RTX 4090 GPU. The implementation was based on the PyTorch2.6.0 framework. During training, the batch size was set to 16, the number of epochs was 100, and the input image resolution was fixed at 640 × 640. To improve model generalization, online data augmentation strategies were applied, including random rotation, scaling transformation, and Mosaic augmentation. To enhance training stability and convergence, Mosaic augmentation was disabled during the final 20 epochs.

All experiments were conducted with fixed random seeds to ensure reproducibility. Unless otherwise stated, the reported results correspond to the checkpoint achieving the best validation mAP@0.9. For the proposed method, repeated runs with different random seeds were additionally performed to evaluate performance stability.

Since the dataset images were extracted from continuous video streams, adjacent frames may exhibit strong temporal similarity. To avoid potential temporal leakage between the training and validation subsets, dataset partitioning was performed at the sequence level rather than at the random frame level. Specifically, frames originating from the same acquisition sequence were grouped together and assigned exclusively to one subset (training, validation, or testing). Therefore, no temporally adjacent frames from the same sequence appeared across different subsets.

Inference speed was measured in frames per second (FPS), defined as the reciprocal of the average inference time per image. All quantitative evaluations were conducted on the validation set, which was verified to contain no temporally correlated frames with the training set based on the SSIM analysis described in Section 2.1. Speed benchmarking was performed with a batch size of 1 to simulate practical online inspection scenarios. All FPS measurements were conducted under identical conditions: batch size = 1, FP32 precision, 200 warm-up iterations, followed by 1000 timed forward passes with torch.cuda.synchronize() enforced at each timing boundary on an NVIDIA RTX 4090 GPU. For all variants incorporating RepBlock, inference-time structural re-parameterization was applied prior to benchmarking, fusing the multi-branch training-time structure into a single equivalent convolution. The higher FPS of the full model relative to lighter intermediate variants is attributed to the simultaneous elimination of two computational bottlenecks: the large-object detection head (removed in P2-2) and the C2f module (replaced by the re-parameterized RepBlock). Their combined effect yields a super-additive reduction in inference latency. During testing, the model was switched to evaluation mode (model.eval()), and gradient computation was disabled using torch.no_grad(). FP16 mixed-precision inference was enabled to further improve computational efficiency

Before formal timing, 100 warm-up iterations were performed to eliminate initialization overhead. The reported FPS corresponds to the average pure forward inference speed measured over the complete test set, excluding data loading time. Image preprocessing and mask postprocessing were evaluated separately. The FPS is calculated as:

$$FPS=\frac{N}{T}$$

where (N) denotes the number of test images and (T) represents the total inference time. Actual deployment speed may vary depending on hardware platform, runtime framework, and whether preprocessing/postprocessing are included.

The task of diamond particle detection requires a careful balance between segmentation accuracy and real-time performance. To comprehensively evaluate the proposed method, multiple quantitative metrics were adopted, including Precision (P), Recall (R), mean Average Precision at a high IoU threshold (mAP@0.9), inference speed, and model complexity.

Considering that particle-size measurement is highly sensitive to boundary accuracy, a stricter IoU threshold of 0.9 was employed instead of the conventional 0.5. This setting emphasizes precise boundary localization and effectively reduces measurement errors caused by segmentation inaccuracies.

Unlike general object detection tasks, the ultimate objective of this study is accurate particle-size quantification rather than coarse object localization. Conventional mAP@0.5 adopts a relatively loose IoU criterion, under which predictions with noticeable boundary deviations may still be regarded as correct. Although such predictions may be acceptable for generic detection tasks, they are insufficient for contour-based size measurement.

In contrast, particle diameter estimation is highly sensitive to mask boundaries, since even minor segmentation errors can propagate to contour perimeter, projected area, and equivalent diameter calculations. Therefore, mAP@0.9 was selected as the primary evaluation metric in this work, as it imposes stricter requirements on boundary alignment and more accurately reflects the segmentation precision required for reliable particle-size analysis.

Furthermore, Precision and Recall were jointly analyzed to characterize the trade-off between false positives and false negatives, thereby ensuring a balanced assessment of detection performance. Model efficiency was further evaluated using the number of parameters and frames per second (FPS), which together reflect computational cost and real-time capability. These metrics provide a comprehensive assessment of the model’s suitability for practical deployment, particularly in scenarios requiring high-throughput and low-latency processing.

Overall, the adopted evaluation framework enables a systematic and objective comparison of both accuracy and efficiency across different models.

To ensure reproducibility, all training runs were conducted using a fixed random seed (seed = 42) applied to the PyTorch2.6.0, NumPy1.19.5, and Python3.9 random modules, with deterministic operations enabled. The reported checkpoint for each model corresponds to the epoch achieving the highest mAP@0.9 on the validation set. For the proposed model, three independent runs with distinct seeds (0, 1, and 2) were additionally conducted to assess training stability. The mean and standard deviation of key metrics are reported in Table 1 alongside the primary results, confirming the consistency and robustness of the reported performance across different initializations.

### 3.2. Particle Size Measurement Evaluation Metrics

The segmentation accuracy measured by mAP@0.9 directly affects the reliability of subsequent particle-size estimation. Therefore, MAE and MAPE are further adopted to quantitatively evaluate the final measurement errors.

To quantitatively evaluate the performance of the proposed diamond particle size measurement method, a set of error-based metrics is adopted, as defined in Equations (12)–(15). To ensure non-negative error representation, absolute values are used in all loss formulations.

Let *DPSCA_i_*, *CADPS_i_* and *MMDPS_i_* denote the particle size of the *i*-th sample obtained by the proposed method, the conventional algorithm, and manual measurement, respectively. The corresponding absolute errors are defined as:

(12) $$Loss\text{\_}1\;=\;\left|DPSCA\;-\;MMDPS\right|$$

(13) $$Loss\text{\_}2=\left|CADPS-MMDPS\right|$$

Based on these definitions, the mean absolute error (MAE) and mean absolute percentage error (MAPE) are calculated as follows:

(14) $$MAE\text{\_}k=\left\{\begin{array}{ll} \frac{1}{n}\sum_{i=1}^{n}|DPSCA_{i}-MMDPS_{i}|, & k=1\\ \frac{1}{n}\sum_{i=1}^{n}|CADPS_{i}-MMDPS_{i}|, & k=2 \end{array}\right.$$

(15) $$MAPE\text{\_}k=\left\{\begin{array}{ll} \frac{100\%}{n}\sum_{i=1}^{n}\frac{\left|DPSCA_{i}-MMDPS_{i}\right|}{MMDPS_{i}}, & k=1\\ \frac{100\%}{n}\sum_{i=1}^{n}\frac{\left|CADPS_{i}-MMDPS_{i}\right|}{MMDPS_{i}}, & k=2 \end{array}\right.$$

where *n* denotes the total number of measured particles.

In this study, the reference measurements (MMDPS) were obtained using the China Liaoning Dandong Baite BT-1600 static image particle analysis system, a commercial particle metrology instrument widely adopted in industrial particle-size characterization. Therefore, it was used as the practical reference standard for quantitative validation.

The conventional algorithm (CADPS) was implemented using OpenCV, including image thresholding, contour extraction, and equivalent diameter computation. This baseline represents low-cost computer vision solutions that are still widely used in industrial practice due to their simplicity and ease of deployment.

Unlike conventional image processing pipelines, the proposed DPSCA framework integrates instance segmentation with contour-based geometric measurement, enabling reliable particle-size estimation under challenging conditions such as particle adhesion, irregular boundaries, and dense distributions. Therefore, the observed performance improvement should be interpreted as the result of the overall integrated measurement framework rather than that of a single geometric estimator.

Although additional algorithmic baselines such as ellipse fitting, watershed-based separation, or alternative segmentation backbones could provide further comparisons, the selected baselines in this study cover both mainstream industrial practice and commercial instrumentation, thereby better reflecting realistic engineering application scenarios.

To ensure the representativeness of the evaluation, diamond particles within the size range of 180–250 μm were selected as experimental samples. This range reflects typical particle characteristics under practical operating conditions and provides a reliable basis for assessing the accuracy and robustness of the proposed measurement approach.

### 3.3. Ablation Studies

**Figure 7:**
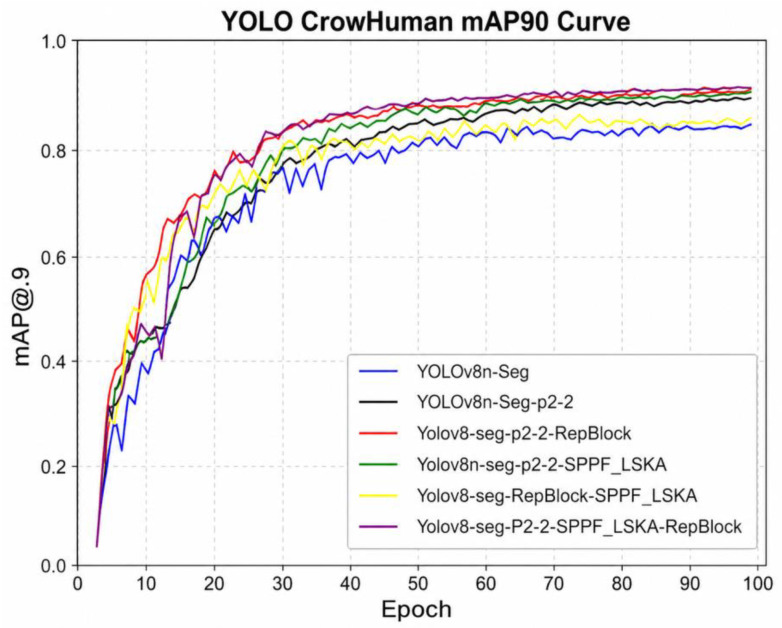
Comparison of mAP@0.9 curves in the improved YOLO model.

Due to the requirement for precise particle-size estimation, mAP@0.9 is adopted as the primary evaluation metric, as it imposes stricter constraints on boundary localization and is more suitable for irregular diamond particles. The results in Figure 7 and Table 1 demonstrate that the proposed improvements consistently enhance model performance. Specifically, the introduction of the P2-2 module significantly reduces the number of parameters (from 3.26 M to 0.79 M) while improving mAP@0.9 to 0.844, indicating that the lightweight design does not compromise accuracy. Further incorporation of the SPPF_LSKA and RepBlock modules enhances feature representation and feature fusion capability, ultimately increasing mAP@0.9 to 0.861.

In terms of efficiency, the optimized model reduces inference time from 3.1 ms to 2.0 ms (approximately 323 FPS to 500 FPS), demonstrating a substantial improvement in real-time performance. Notably, all models achieve comparable performance under mAP@0.5 (>0.895), whereas mAP@0.9 more effectively highlights differences in localization accuracy. Overall, the proposed modules complement each other by improving high-resolution feature representation, expanding the receptive field, and enhancing feature fusion, enabling the model to achieve a favorable trade-off between accuracy and computational efficiency.

### 3.4. Comparative Experiments

**Figure 8:**
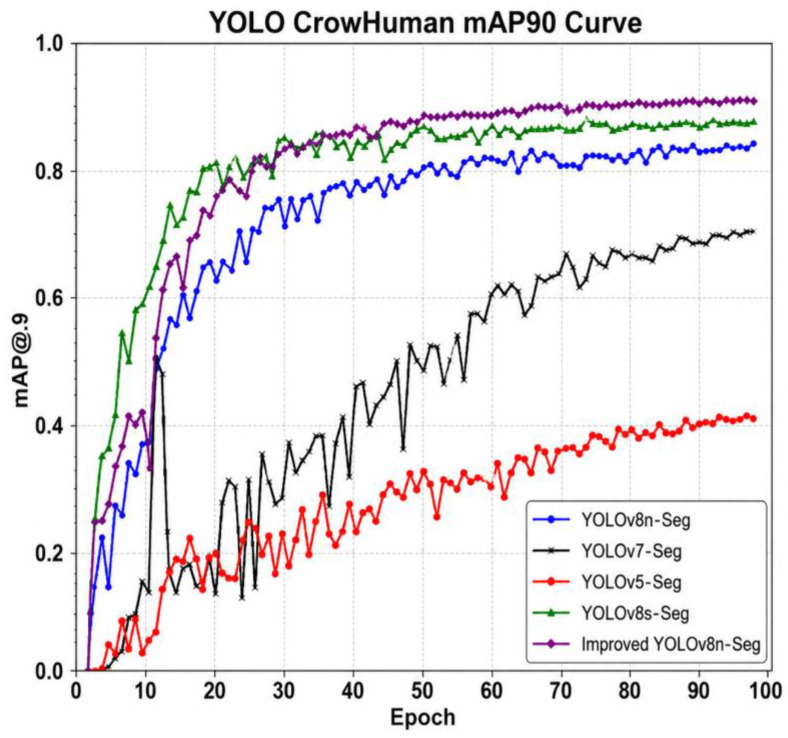
Comparison of mAP@0.9 curves for different YOLO models.

In this section, several representative models are compared, including YOLOv5n-Seg [23], YOLOv7-Seg [24], YOLOv8n-Seg [25], YOLOv8s-Seg, Mask R-CNN [26], YOLOv11-Seg [27], and the proposed improved YOLOv8n-Seg. All models are fully trained under the same experimental conditions. The comparative results are illustrated in Figure 8 and summarized in Table 2.

As shown in the results, the improved YOLOv8n-Seg achieves the best overall performance among all evaluated models. In particular, its mAP@0.9 reaches 0.861, which is 6.2% higher than the baseline YOLOv8n-Seg (0.799), demonstrating its superiority in high-precision segmentation tasks. Although YOLOv5n-Seg exhibits relatively fast inference speed due to its lightweight architecture, its mAP@0.9 is only 0.402, making it unsuitable for accurate particle boundary detection. In contrast, Mask R-CNN achieves the highest mAP@0.5 (0.950), but its performance decreases to 0.835 under the stricter mAP@0.9 metric, while also incurring substantially higher computational complexity (43.98 M parameters and lower FPS).

In terms of efficiency, the proposed model reduces the parameter count to 0.97 M, which is approximately 70% lower than the baseline YOLOv8n-Seg, while increasing inference speed to 500 FPS (2 ms per image). Compared with YOLOv8s-Seg and YOLOv11-Seg, which achieve competitive accuracy but at significantly higher computational cost, the proposed method provides a superior trade-off between accuracy and efficiency.

Overall, the improved YOLOv8n-Seg demonstrates clear advantages in precise boundary localization, lightweight design, and real-time performance, making it particularly suitable for dense, small-scale diamond particle detection tasks.

### 3.5. Boundary Fidelity and Measurement Correlation Analysis

Although mAP@0.9 provides a stricter overlap-based evaluation than the conventional mAP@0.5, it does not directly quantify contour fidelity or the practical impact of segmentation errors on particle-size estimation. Therefore, additional boundary-sensitive metrics were introduced in this study, including the Dice coefficient and Boundary IoU.

The Dice coefficient measures the similarity between predicted masks and ground-truth annotations, while Boundary IoU specifically evaluates contour alignment quality. As shown in Table 3, the proposed SPR-YOLOv8 achieves the best overall performance among all compared methods, indicating superior mask integrity and boundary localization capability.

These results confirm that the advantages of the proposed framework are not limited to conventional detection metrics, but also translate into more reliable performance in downstream particle metrology tasks.

### 3.6. Visualization of Test Results

**Figure 9:**
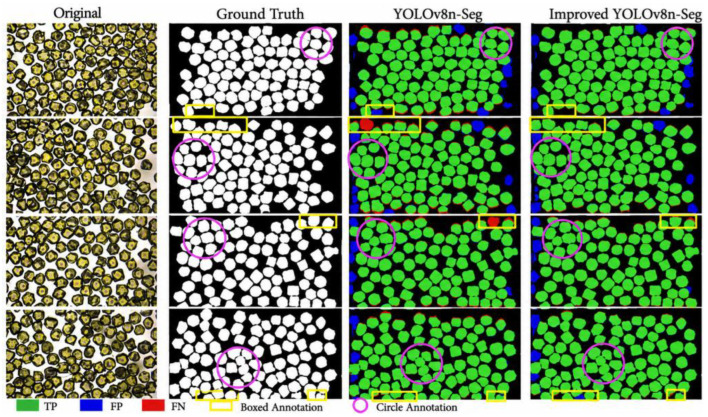
Comparison of segmentation results before and after improvement.

To visually demonstrate the effectiveness of the improved YOLOv8n-Seg model, qualitative comparisons are conducted on the same test set, as shown in Figure 9. Compared with the baseline model, the proposed method achieves more accurate boundary localization while maintaining a high true positive rate. In densely distributed regions, the improved model can effectively distinguish adjacent particles, significantly reducing both over-segmentation and under-segmentation errors. This observation is consistent with the slightly higher Recall value, indicating improved detection completeness.

Moreover, the baseline model tends to produce false positives in inter-particle gaps, whereas the improved model effectively suppresses such errors, which can be attributed to the enhanced feature representation introduced by the SPPF_LSKA module. As highlighted in the marked regions, the improved model produces smoother and more continuous segmentation boundaries, preserving the geometric integrity of diamond particles more effectively. This is particularly important for particle-size measurement, where boundary accuracy directly influences the reliability of quantitative analysis.

Across different scenarios, including dense, sparse, and boundary regions, the improved YOLOv8n-Seg demonstrates stable and robust performance. It accurately separates closely packed particles, avoids false detections in sparse regions, and maintains reliable segmentation near image edges. Overall, the visualization results are consistent with the quantitative evaluations, confirming that the proposed method significantly improves segmentation accuracy and robustness, making it well suited for precise particle-size measurement in complex environments.

To preliminarily evaluate the robustness of the proposed framework under domain shifts caused by particle-scale variation, additional experiments were conducted on unseen particle batches with nominal size ranges of 25–40 μm and 250–350 μm. These supplementary samples were not involved in model training or hyperparameter tuning.

**Figure 10:**
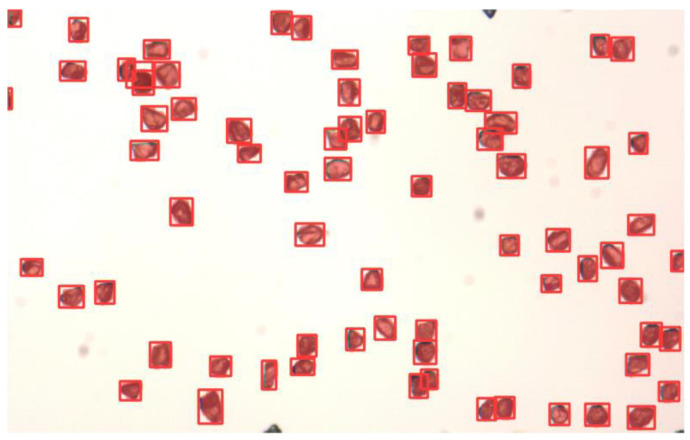
Qualitative cross-scale generalization results on 25–40 μm diamond particles. The red box represents the recognized particles.

For the 25–40 μm samples, despite the substantially smaller object size and higher spatial density, the model successfully detected most visible particles with high confidence, as shown in Figure 10, indicating that the P2-2 high-resolution detection head effectively enhances sensitivity to micro-scale targets.

For the 250–350 μm samples, the model also maintained stable segmentation performance, demonstrating that the proposed multi-scale feature representation generalizes well to relatively larger particles.

Overall, these results suggest that the proposed framework exhibits promising cross-scale transfer capability beyond the primary 180–250 μm dataset.

### 3.7. Particle Size Calculation Algorithm Evaluation

In this study, diamond particles with a target size range of 180–250 μm were selected, consistent with commonly used industrial specifications. Two representative images were randomly sampled for comparative analysis, containing 25 and 33 particles, respectively. The particle sizes were sorted and statistically analyzed, and the results are summarized in Table 4.

Compared with the manual measurement reference (MMDPS), the conventional algorithm (CADPS) exhibits relatively larger errors. Specifically, the mean absolute error (MAE) reaches 1.22 μm and 1.47 μm for the two samples, while the mean absolute percentage error (MAPE) is 0.57% and 0.69%, respectively. These results indicate the presence of missed detections and inaccurate boundary extraction, which adversely affect measurement reliability.

In contrast, the proposed DPSCA method demonstrates significantly improved accuracy. The MAE values are reduced to 0.27 μm and 0.41 μm, while the MAPE values decrease to 0.13% and 0.19%, respectively. This substantial reduction in error highlights the effectiveness of the proposed method in capturing precise particle boundaries. Moreover, the consistent performance across samples with different particle counts indicates strong robustness and stability under varying particle distribution conditions.

Overall, these results further demonstrate the superiority of the proposed method for high-precision particle-size measurement in practical applications.

**Figure 11:**
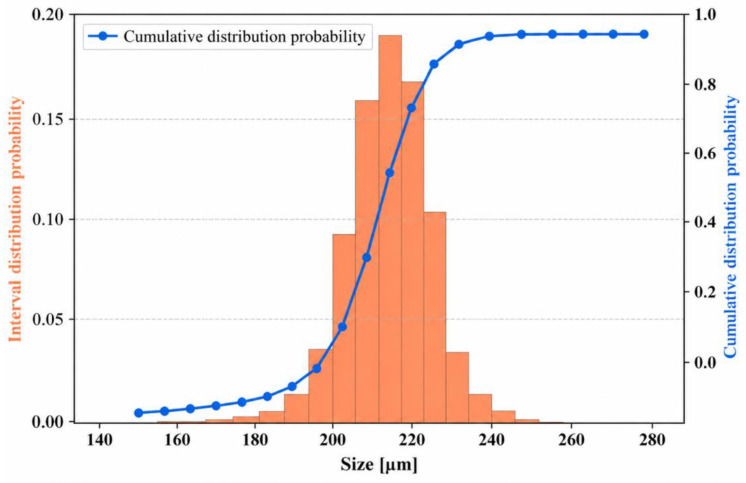
Diamond particle size distribution chart.

Furthermore, a large-scale experiment involving 8890 diamond particles is conducted to validate the statistical reliability of the proposed method. The particle size distribution and cumulative distribution are illustrated in Figure 11, showing that most particles are concentrated within the range of 200–230 μm, which is consistent with the target range of 180–250 μm. This confirms the effectiveness of the sampling and measurement approach. For dynamic analysis, a particle tracking strategy based on IoU and centroid distance is employed, where particles with IoU > 0.6 and centroid displacement <20 pixels between adjacent frames are considered the same instance. Experimental results demonstrate a tracking accuracy of 96.4%, an ID switch rate below 3.2%, and a size consistency error below 2.8% across consecutive frames, indicating that the proposed method satisfies the stability requirements for real-time video-based particle size measurement.

The results indicate that accurate particle-size analysis depends not only on segmentation precision, but also on robust contour interpretation and geometry-consistent size estimation. This validates the effectiveness of the proposed integrated DPSCA framework.

## 4. Conclusions

In this study, we propose and validate a novel integrated computer vision system for real-time diamond particle segmentation and size analysis under dynamic industrial conditions. The system consists of three synergistic components: (1) a fully automated image acquisition platform that captures continuous video streams of particles, providing realistic and high-quality data; (2) the SPR-YOLOv8 model, a lightweight instance segmentation network specifically optimized for small and densely distributed targets through the integration of the SPPF_LSKA module, RepBlock, and a P2-2 detection head; and (3) the DPSCA, which converts segmentation masks into accurate particle-size measurements based on contour geometry.

The primary strength of the proposed system lies in its balanced and superior overall performance. The SPR-YOLOv8 model serves as the core component, achieving a high-precision mAP@0.9 of 0.861 while maintaining an inference speed of 500 FPS (2 ms per image) and a compact model size of only 0.97 M parameters. This efficiency enables real-time processing without compromising accuracy. More importantly, the end-to-end performance of the complete system was rigorously validated. Compared with conventional image processing methods (CADPS), the proposed system (DPSCA-based measurement) reduces the mean absolute percentage error (MAPE) by over 70%, demonstrating strong agreement with professional instrument measurements (MMDPS). The system also exhibits robust performance in dynamic tracking scenarios.

Overall, this work goes beyond algorithmic improvement and delivers a practical, reliable, and fully automated inspection solution. It effectively addresses the limitations of manual operation and traditional machine vision methods in diamond particle sizing, offering significant potential for online quality control in superhard material manufacturing. Preliminary experiments on 25–40 μm and 250–350 μm particle batches further indicate promising transferability under scale variations. Future work will focus on broader domain adaptation across different suppliers, material types, imaging optics, illumination conditions, and highly occluded industrial environments.

## Figures and Tables

**Table 1:** Comparison of ablation test results.

Model	Precision	Recall	mAP@0.5	mAP@0.9	mAP@.5:95	Parameters	FPS
Baseline	0.787	0.873	0.897	0.799	0.816	3,258,454	180
+repblock	0.786	0.861	0.897	0.805	0.817	4,117,462	155
+P2-2	0.761	0.878	0.891	0.844	0.837	785,140	310
+p2-2 + LSKA	0.782	0.868	0.898	0.852	0.842	856,052	290
+p2-2 + repblock	0.791	0.853	0.895	0.854	0.839	925,524	270
+LSKA + repblock	0.797	0.849	0.898	0.803	0.818	458,0230	145
Ours (Full Model)	0.778	0.874	0.898	0.861	0.842	972,460	500

**Table 2:** Comparison of experimental results. All models were evaluated under a single training run with a fixed random seed (seed = 42).

Model	Precision	Recall	mAP@0.5	mAP@0.9	mAP@.5:95	Parameters	FPS
YOLOv5n-Seg	0.609	0.826	0.744	0.402	0.597	1,881,103	210
YOLOv7-Seg	0.786	0.855	0.893	0.669	0.783	37,847,870	55
YOLOv8n-Seg	0.787	0.873	0.897	0.799	0.816	3,258,454	180
YOLOv8s-Seg	0.809	0.863	0.911	0.828	0.835	11,790,854	110
Mask R-cnn	0.782	0.861	0.950	0.835	0.765	43,980,000	45
YOLOv11-Seg	0.795	0.868	0.905	0.82	0.83	22,340,000	80
Ours(Full Model)	0.778	0.874	0.898	0.861	0.842	972,460	500

**Table 3:** Comparison of boundary-sensitive metrics.

Model	mAP@0.9	Dice	Boundary Iou	MAE (μm)
YOLOv5n-Seg	0.402	0.861	0.732	1.22
YOLOv8n-Seg	0.799	0.908	0.846	0.61
YOLOv8s-Seg	0.828	0.919	0.861	0.54
Mask R-cnn	0.835	0.924	0.872	0.48
Ours(Full Model)	0.861	0.936	0.891	0.27

**Table 4:** Comparison of individual diamond particle size measurements.

Number	*MMDPS*	*DPSCA*	*CADPS*	*Loss*_1	*Loss*_2
1	235.88	236.1	239.99	0.22	3.47
2	229.74	229.43	231.85	0.31	2.88
3	227.11	226.72	228.95	0.39	1.52
4	222.57	222.41	223.88	0.16	0.42
5	221.5	221.61	223.45	0.11	0.92
6	220.98	220.72	222.5	0.26	1.49
7	220.65	220.41	221.89	0.24	0.83
8	220.01	219.83	220.11	0.18	0.09
9	219.55	219.39	220.06	0.16	0.3
10	218.67	218.48	219.35	0.19	0.88
11	217.89	217.61	219.17	0.28	1.4
12	216.47	216.32	217.99	0.15	0.93
13	214.64	214.29	216.51	0.35	1.83
14	211.72	211.48	212.77	0.24	0.89
15	210.8	210.62	211.62	0.18	0.73
16	209.42	209.15	210.56	0.27	0.55
17	208.67	208.42	210.19	0.25	1.79
18	208.21	207.89	209.78	0.32	1.65
19	207.98	207.72	209.5	0.26	1.54
20	207.31	206.98	209.22	0.33	2.1
21	200.35	200.12	200.91	0.23	0.43
22	198.88	198.59	199.77	0.29	0.44
23	197.58	197.39	198.55	0.19	0.77
24	190.76	190.11	191.73	0.65	1.28
25	186.4	185.82	188.1	0.58	1.29
MAE_k				0.27	1.22
MAPE_k				0.13%	0.57%
1	236.75	237.25	240.65	0.5	3.9
2	230.31	230.16	232.27	0.15	1.96
3	228.53	227.19	227.83	1.34	0.7
4	222.89	222.65	223.31	0.24	0.42
5	221.01	221.07	222.64	0.06	1.63
6	220.89	220.96	222.57	0.07	1.68
7	220.78	220.85	221.56	0.07	0.78
8	220.61	220.51	220.58	0.1	0.03
9	220.41	219.97	220.39	0.44	0.02
10	219.71	219.47	220.08	0.24	0.37
11	218.23	217.86	219.92	0.37	1.69
12	217.11	217.2	219.79	0.09	2.68
13	216.98	216.72	218.54	0.26	1.56
14	214.14	213.69	217.38	0.45	3.24
15	214.12	213.37	216.88	0.75	2.76
16	212.23	211.83	213.97	0.4	1.74
17	211.88	211.76	212.86	0.12	0.98
18	210.97	210.71	211.88	0.26	0.91
19	209.88	209.44	210.63	0.44	0.75
20	208.16	208.01	210.55	0.15	2.39
21	208.05	207.47	210.25	0.58	2.2
22	207.88	207.22	210.06	0.66	2.18
23	207.49	207.07	209.93	0.42	2.44
24	207.35	206.93	209.02	0.42	1.67
25	206.55	206.02	208.87	0.53	2.32
26	205.03	205.37	206.57	0.34	1.54
27	203.01	203.31	202.95	0.3	0.06
28	201.42	201.39	201.86	0.03	0.44
29	198.35	198.24	198.58	0.11	0.23
30	197.69	196.91	198.3	0.78	0.61
31	196.88	196.58	197.92	0.3	1.04
32	195.01	195.87	193.45	0.86	1.56
33	187.22	185.63	189.31	1.59	2.09
MAE_k				0.41	1.47
MAPE_k				0.19%	0.69%

## Data Availability

The raw data supporting the conclusions of this article will be made available by the authors on request.

## Abbreviations

The following abbreviations are used in this manuscript:

IOU	Intersection and Union
μm	Micrometer
Dice	Dice Similarity Coefficient
mAP	mean Average Precision
FPS	Frames Per Second
LSKA	Large Separable Kernel Attention
RepBlock	Re-parameterization Block
